# Sertaconazole versus Clotrimazole and Miconazole Creams in the Treatment of Otomycosis: A Placebo-Controlled Clinical Trial 

**DOI:** 10.22038/IJORL.2021.54805.2872

**Published:** 2022-01

**Authors:** Shadman Nemati, Hooshang Gerami, Ali Faghih Habibi, Ehsan Kazemnejad, Noushin Shabani, Vahid Aghsaghloo, Sina Montazeri

**Affiliations:** 1 *Otorhinolaryngology Research Center, School of Medicine, Guilan University of Medical Sciences, Rasht, Iran.*

**Keywords:** Clotrimazole, Miconazole, Otitis Externa, Otomycosis, Sertaconazole

## Abstract

**Introduction:**

Fungal otitis extern or otomycosis, is common worldwide, and resistance of fungal organisms to antifungal drugs has been reported in otomycosis and other fungal infections. This study aimed to evaluate the clinical efficacy of sertaconazole versus placebo, as well as miconazole and clotrimazole topical creams in otomycosis patients.

**Materials and Methods:**

In this double-blinded clinical trial, 138 otomycosis patients (230 ears) were evaluated in four groups. After the first session of the ear canal debridement and irrigation with acetic acid 2% solution, the patients were treated with either A) sertaconazole 2% cream, B) miconazole 2% cream, C) clotrimazole 2% cream, or D) placebo. The results of clinical evaluations and response to treatment (complete, partial, and no response) were recorded at the time of the first visit and by the end of the first, second, and fourth weeks of treatment. A p-value less than 0.05 was considered statistically significant.

**Results:**

Response results to treatments, ear itching, aural fullness, otalgia, and otorrhea revealed significant differences in either group A or groups B and C, compared to the placebo group (P<0.05). Considering both complete and partial responses together, the sertaconazole group showed a 96.43% response rate. For complete response, miconazole revealed better results, compared to the other two creams; however, the differences for the therapeutic outcomes were not statistically significant. No adverse reactions were observed in the study groups.

**Conclusions:**

Sertaconazole had comparable results with miconazole and clotrimazole in the treatment of otomycosis, and especially if complete and partial responses were considered together, it was more efficacious than miconazole and clotrimazole creams.

## Introduction

Otomycosis or fungal otitis extern accounts for about 15%-20% of the cases with external otitis worldwide that is more frequently observed in humid or tropical regions, such as north of Iran (1-5). Aspergillosis and Candida species are the major pathogens (3, 5). The clinical manifestations often include ear itching, otalgia, otorrhea, and hearing loss. In most cases, white or black creamy discharges in the external auditory canal (EAC) on the otoscopic examination lead to a definite diagnosis (4-8). Various therapeutic approaches, such as pathogen-specific antifungal medications with maintaining aural hygiene are applied in the treatment of otomycosis. Azole class medications, such as clotrimazole and miconazole (in the forms of drops or creams), are effective in the treatment of otomycosis (6-8). Miconazole has been used for over 30 years in patients with superficial fungal infections (9-13). 

Sertaconazole, like other imidazole antifungals, blocks the synthesis of ergosterol, and then inhibits peroxidase accumulation in the cells; however, it also contains a benzothiophene ring which increases the drugs' ability to form pores in the fungal cell membrane, and the fungal cell will die because of losing adenosine triphosphate through these pores. Sertaconazole is thought to be the only imidazole antifungal with this mechanism of action, and it also has anti-inflammatory, antipruritic, and antibacterial actions. Candida albicans, Epidermophyton floccosum, Trichophyton mentagrophytes, and Trichophyton rubrum are known fungal species that have been shown to be susceptible to sertaconazole in previous studies (14-18). This medication is often used in the management of superficial mycoses in dermatology and gynecology, such as athlete's foot and fungal vaginitis (15-17), and to the best of our knowledge, there are limited studies, including a study conducted by Sumbria D. et al. (18) that investigated sertaconazole cream efficacy in otomycosis cases. Resistance of fungal organisms to nystatin, ketoconazole, clotrimazole, and other antifungal drugs has been reported in otomycosis and other fungal infections (5-10,13-15,18,19), and investigating the efficacy of different forms of these drugs in otomycosis cases in various regions and countries is indicated. Furthermore, there are very few studies that compare the efficacy and safety profiles for the various antifungal agents, and as such, there is no clear consensus among otolaryngologists about the relative treatment efficacy of these preparations (20). On the other hand, plenty of studies about the efficacy of these drugs on fungal species are *in vitro*, and few studies have compared the "clinical" efficacy of sertaconazole cream with two other more commonly prescribed antifungal creams and placebo in otomycosis patients. Therefore, the present study aimed to compare the clinical efficacy of sertaconazole, miconazole, and clotrimazole creams with each other and with a placebo in a sample of 230 patients with otomycosis. 

## Materials and Methods

A total of 230 ears with clinical signs and symptoms of otomycosis were enrolled in this prospective double-blinded clinical trial. The study protocol was approved by the Guilan University of Medical Sciences at Otorhinolaryngology Research Center and also by the Ethical Committee of Guilan University of Medical Sciences (1930459701). The proposal of this study was registered in Iranian Registry of Clinical Trials (IRCT2015022411 38N15).

The diagnosis of otomycosis for each patient was made by an expert university otolaryngologist based on the disease history (i.e., external ear itching, otalgia, ear fullness), physical examinations (microscopic otoscopy of external auditory canals), and classical oto-microscopic manifestations (dry-whitish furry debris with or without blackish spores, or wet-pasty/creamy debris resembling wet newspaper along with signs of inflammation in the canal). Cultures from the ear discharges were not obtained in this study. Written informed consent was obtained from all subjects; moreover, a thorough explanation about the study was given to the patients, and they were free for leaving the study whenever they want. All the data remained completely confidential. Furthermore, a detailed checklist was filled out for each subject, including the demographic characteristics (age, gender), and patients' clinical manifestations (e.g., external auditory canal itching, otorrhea, otalgia, aural fullness sensation, and hearing loss). 

At first, numbers from one to 148 were written on the pieces of paper with the order of A, B, C, and D (group names) on the back of the papers. Subsequently, these papers were placed in a box and handed to the secretary in charge of admissions at the otolaryngology clinic of Amiralmomenin hospital, Rasht, Iran; so that any patient with otomycosis could pick up a number from the box, determining his/her treatment group. After a thorough description of the study from otolaryngology attending and residents, the referred patients with otomycosis volunteering to participate in the trial were introduced to those secretaries. The exclusion criteria were: 1) external otitis with external auditory canal stenosis, 2) chronic otitis media, perforated tympanic membrane, and chronic discharging ears, 3) positive history for previous ear surgeries, diabetes mellitus, and immune deficiency conditions, and 4) positive history of hypersensitivity to azole drugs. Any possible complications and drug reactions were closely monitored and managed in this study. 

All patients underwent oto-microscopic examination, and debridement of EAC and irrigation of the EAC with acetic acid 2% solution during debridement were two important parts of their treatment process. In addition, all cases were asked to keep their ears dry. Indeed, all of the cases received the mainstay of antifungal treatment, and then, they were categorized into four groups receiving: A) sertaconazole 2% topical cream (Dermofix, Ferrer, Actoverco), B) miconazole 2% topical cream (Behvazan Bio-Pharmaceuticals), C) clotrimazole 2% topical cream (Behvazan Bio-Pharmaceuticals), and D) placebo (topical vitamin A+D cream).

There are many topical antifungal and antiseptics, as well as acidifying agents, for the treatment of otomycosis (20); however, to the best of our knowledge, there is not any reference supporting the antifungal or even antiseptic effects of topical vitamin D and A creams till now. Accordingly, they were considered placebos. 

All the creams were applied using a small 2cc syringe and 18-gauge intravenous catheter by an otolaryngologist to assure the proper and easy administration of the medications for the patients. The applied creams were removed from the EAC one week later along with a microscopic inspection of the canal and clinical evaluation of the response to the treatment by another otolaryngologist that was blinded to the patients' groups. Afterward, the patients were referred to the secretary again, and her/his therapeutic cycle was repeated for another week with the first otolaryngologist and the same initial creams. At the end of the second and fourth weeks, after the initial administration of the creams, the patients underwent EAC re-inspection, evaluating their clinical responses to the treatment. 

The clinical responses were classified as a complete or good response (i.e., patients with minimally or no symptoms, as well as dry EAC and tympanic membrane [TM] with no discharge remnants in oto-microscopy), partial or moderate response (i.e., moderate symptoms and minimal secretions in EAC or on the TM), and no response (i.e., persistent severe symptoms and excess amounts of fungal discharges in EAC or on the TM). 

It is worth mentioning that contrary to the patients and the second otolaryngologist who performed follow-up visits, the secretary and the first otolaryngologist were not blinded to the applied interventions.

All data were analyzed in SPSS software (version 21.0). Man-Whitney and Wilcoxon statistical tests were applied to compare EAC itching among the three groups and evaluate this factor before and after the treatment. Furthermore, the Chi-square and Cochran tests were utilized to compare auditory canal fullness sensation, otalgia, otorrhea, and response to treatments among the three groups. A p-value less than 0.05 was considered statistically significant.

## Results

This study included 148 patients with otomycosis; however, 138 patients (230 ears) finally completed the study course (five patients in the placebo group, one patient in group A, as well as two patients in groups B and C failed to complete the follow-up course). In assessing the demographic characteristics (Table 1), the majority of the patients (46.96%) were 30-50 years old, and the differences among the groups were not statistically significant (P=0.237). Moreover, the majority of the patients among the four groups had high school diplomas (P=0.048), and more than 66% of the cases had bilateral ear involvements.

**Table 1:** Demographic characteristics (i.e., gender, age, general education level, and side of affected ears) of the patients in the four groups of the study

**Variables Groups**			**Placebo (n=58)**	**Clotrimazole** **(n=58)**	**Miconazole** **(n=58)**	**Sertaconazole** **(n=56)**	**Total (n=230)**	**P-Value**
Gender	Male	N (%)	28(48.28)	33(56.90)	27(46.55)	24(42.86)	112(48.69)	0.572*
	Female	N (%)	30(51.72)	25(43.10)	31(53.45)	32(57.14)	118(51.30)	
Age ( y.o)	<30	N (%)	16(27.59)	21(36.21)	12(20.69)	10(17.86)	59(25.65)	0.237*
	30-50	N (%)	31(53.45)	21(36.21)	30(51.72)	26(46.43)	108(46.96)	
	>50	N (%)	11(18.97)	16(27.59)	16(27.59)	20(35.71)	63(27.39)	
	Mean(±SD)		37.19(11.40)	37.72(15.23)	40.81(11.48)	44.50(15.73)	39.40(13.40)	
Education level	Undergraduate	N (%)	4(6.90)	16(27.59)	11(18.97)	28(50.00)	59(25.65)	<0.001*
	High school diploma	N(%)	29(50.00)	24(41.38)	30(51.72)	22(39.29)	105(45.65)	
	University graduate	N(%)	25(43.10)	18(31.03)	17(29.31)	6(10.71)	66(28.69)	
Ear	Right	N(%)	6(10.34)	5(8.62)	4(6.90)	24(42.86)	39(16.95)	<0.001*
	Left	N(%)	5(8.62)	2(3.45)	6(10.34)	26(46.43)	39(16.95)	
	Both	N(%)	47(81.03)	51(87.93)	48(82.76)	6(10.71)	152(66.08)	

*chi-square test

N: number, %: percent, SD: Standard Deviation

Table 2 shows the clinical manifestations of the patients with otomycosis in the first visit and throughout the three follow-up visits. All the patients in the groups had ear itching before the study, and 97.39% of the cases had severe ear itching. There was not a significant difference among the groups in terms of otorrhea and otalgia (P>0.05). After the first week of treatment, otalgia, ear itching, otorrhea, and response to treatments revealed significant changes among all treatment groups, compared to the placebo group (P=0.0001, P=0.002, and P=0.0001, respectively). 

**Table 2:** Clinical manifestations of the patients with otomycosis in the first visit and throughout three follow-up visits

**P-Value**	**Week 4**	**Week 2**	**Week 1**	**Week 0**	**Symptoms** ** Weeks** **of treatment**		
*<0.001	20(35.71)	34(60.71)	39(69.64)	50(89.29)	N(%)	Sertaconazole	Ear itching
<0.001*	25(43.10)	35(60.34)	41(70.69)	58(100.00)	N(%)	Miconazole	
0.002*	15(25.86)	31(53.45)	29(50.00)	58(100.00)	N(%)	Clotrimazole	
0.230*	46(79.31)	40(68.97)	48(82.76)	58(100.00)	N(%)	Placebo	
<0.001*	1(1.79)	6(10.71)	12(21.43)	48(85.71)	N(%)	Sertaconazole	Aural fullness
<0.001*	0.0(0.0)	5(8.62)	10(17.24)	45(77.59)	N(%)	Miconazole	
<0.001*	2(3.45)	6(10.34)	12(20.69)	47(81.03)	N(%)	Clotrimazole	
0.067*	33(56.90)	26(44.83)	20(34.48)	36(62.07)	N(%)	Placebo	
<0.001*	2(3.57)	4(7.14)	22(39.29)	54(96.43)	N(%)	Sertaconazole	Otorrhea
<0.001*	3(5.17)	8(13.79)	18(31.03)	57(98.28)	N(%)	Miconazole	
<0.001*	7(12.07)	12(20.69)	25(43.10)	57(98.28)	N(%)	Clotrimazole	
0.122*	47(81.03)	44(75.86)	45(77.59)	56(96.55)	N(%)	Placebo	
<0.001**	0.0(0.0)	1(1.79)	2(3.57)	16(28.57)	N(%)	Sertaconazole	Otalgia
<0.001**	0.0(0.0)	0.0(0.0)	0.0(0.0)	16(27.59)	N(%)	Miconazole	
<0.001**	1(1.72)	1(1.72)	1(1.72)	19(32.76)	N(%)	Clotrimazole	
0.048**	12(20.69)	6(10.34)	3(5.17)	18(31.03)	N(%)	Placebo	

*chi-square test-

**Cochran test

N: number, %: percent

Outcomes at the end of the second and fourth weeks of the treatments have been provided in Table 2. In the sertaconazole group, ear itching was relieved mostly on the fourth week of the treatment; however, other symptoms, including otalgia, otorrhea, and aural fullness, relieved significantly on the first and second weeks and obviously on the fourth week. It seems that only clotrimazole cream revealed a good effect on ear itching at the end of the first week, and the good effects of sertaconazole cream on ear itching and otorrhea took at least two weeks to be revealed.

**Table 3:** *Response to treatments in different treatment groups and follow-up visits; 'Yes' means good/ complete response, and 'Partial' means relatively good response*

**P-Value**	**Week 4**	**Week 2**	**Week 1**	** Treatment/Response Weeks**		
<0.001*	50(89.29)	46(82.14)	34(60.71)	N(%)	Yes	Sertaconazole
	2(3.57)	0.0(0.0)	10(17.86)	N(%)	No	
	4(7.14)	10(17.86)	12(21.43)	N(%)	Partial	
<0.001*	55(94.83)	50(86.21)	38(65.52)	N(%)	Yes	Miconazole
	3(5.17)	6(10.34)	17(29.31)	N(%)	No	
	0.0(0.0)	2(3.45)	3(5.17)	N(%)	Partial	
<0.001*	51(87.93)	45(77.59)	33(56.90)	N(%)	Yes	clotrimazole
	5(8.62)	7(12.07)	15(25.86)	N(%)	No	
	2(3.45)	6(10.34)	10(17.24)	N(%)	Partial	
0.23	10(17.24)	12(20.69)	10(17.24)	N(%)	Yes	placebo
	12(20.69)	17(29.31)	14(24.14)	N(%)	No	
	36 (62.07)	29(50)	34(58.62)	N(%)	Partial	

*Chi-square test

**Fig 1:**
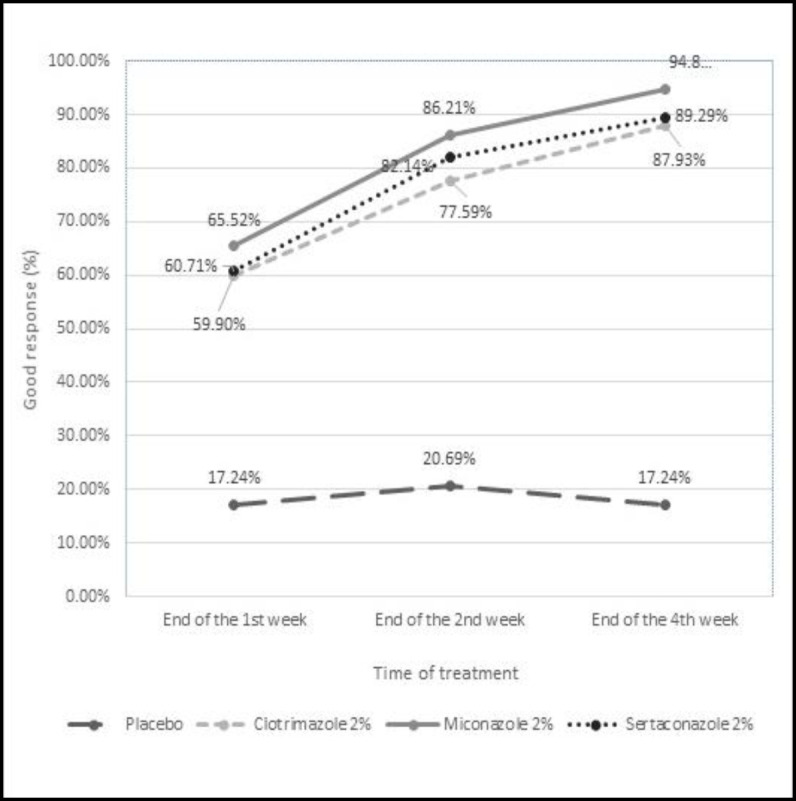
"Complete or good response" rates of the four study groups to treatment medications

**Fig 2:**
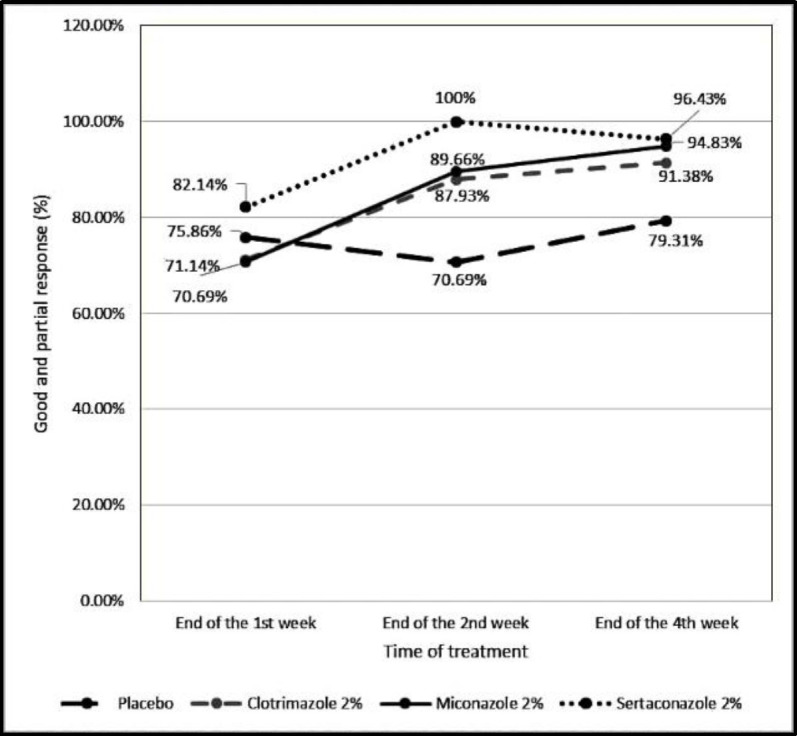
"Complete (good) and/or partial (relatively good) response" rates in patients taking the medications in the four groups

Figures 1 and 2, as well as Table 3, show the responses to the treatments in different groups in different follow-up visits. Good or complete responses to the treatments in the sertaconazole group were obtained at 60.71%, 82.14%, and 89.29% at the end of the first, second, and fourth weeks, respectively. Final results at the end of the fourth week revealed that complete responses were presented for 55 (94.8%) ears in the miconazole group, compared to 50 (89.3%) ears in the sertaconazole group, 51 (87.9%) ears in the clotrimazole group, and 10 (17.24%) ears in the placebo group (Table 2). 

Considering complete and partial response together, as the positive response to the treatments, the sertaconazole group showed a 96.43% response rate, compared to 94.83%, 91.38%, and 79.31% response rates for miconazole, clotrimazole, and placebo groups, respectively (P=0.02) (Figures 1,2). 

No adverse reactions were reported or observed in our study groups.

## Discussion

Otomycosis is a common fungal infection in otology clinics, mostly presented with ear itching, otalgia, otorrhea, and hearing loss. As a frequent disease in humid environments, it is commonly found in the northern regions of Iran (4,5,13). The diagnosis is usually made based on the patients' history and physical examinations. Despite various treatment regimens, including local debridement, acidifying ear canal, and/or antifungal agent administration, ongoing research is still being conducted worldwide to decrease the disease duration and its burden. 

Like other treatments, the treatment of otomycosis is affected significantly by the microorganisms' resistance against the antibiotics and antifungal agents and by the low compliance of the patients in administering the physicians' orders and using oto-topical antifungal drops incorrectly or incompletely. Therefore, many ear, nose and throat (ENT) specialists encounter repeating otomycosis and failure of treatment because of these reasons. In this regard, it is thought that the treatment of otomycosis by topical creams that are mainly administered by the physician in a weekly dosage may be superior to administering topical drops in several time-daily dosages and by the patients. In this study, the efficacy of sertaconazole 2% cream was evaluated, and its effects were compared on otomycosis with two commonly prescribed topical creams (miconazole and clotrimazole 2%) and with a placebo. Previous studies have focused on both the fungal growth spectrum and the therapeutic methods or *in vitro* anti-fungal sensitivity rates (5). However, as the diagnosis and treatment of otomycosis does not require culturing or/and defining the type of fungal elements, the subjects were enrolled in our study groups without fungal culturing and defining the type of causative fungi.

Almost half of our patients were men (48.69%), while the male to female ratio was 1:4 in a study by Pontes et al. (14). However, 65.11% and 53% of the subjects in the studies by Nemati et al. and Satish et al. were women, respectively (5,13). Furthermore, Kiakojouri et al. reported more women having otomycosis than men (11). Most of the cases in our study were aged 30-50 years old (y/o), while the age range varied from 21 to 30 y/o in a study by Satish et al. (13) and from 27 to 48 y/o in other studies (12-14). This is probably due to various working environments, including dusty surroundings with poor hygiene standards (5). 

Additionally, according to former studies (6-9,18), the involvement of both ears with otomycosis was not common; however, consistent with the results of a study by Pontes et al., otomycosis was observed in both ears of more than 66% of the subjects in all of the study groups. Moreover, contrary to the findings of a study by Sumbria et al. (18) who reported the right ear involvement more frequently by otomycosis, in our patients, right and left ears involved equally. Severe itching of ears was the most frequent complaint of all patients (97.39%) at the beginning of our study. This finding is also similar to the results of the studies by Kiakojouri et al. and Pontes et al., reporting the itching of the ears in 86.4% and 60% of the patients, respectively (11,14). On the other hand, Alnawaish et al. reported otalgia as the most common symptom in 50 (55%) patients (12), while only 30% of our subjects had otalgia at the beginning of the study. In a study conducted by Sumbria et al. (18), otalgia with ear itching were the most (50.7%) common presenting symptoms, followed by itching alone in 17.8%, otalgia, itching, and ear discharge in 11.8%, as well as itching and ear discharge in 7.9% of the subjects.

As with treatment options for acute bacterial otitis extern, there are very few studies that compare the efficacy and safety profiles for the various antifungal agents. As such, there is no clear consensus among otolaryngologists about the relative treatment efficacy of these preparations (20). Especially, there are few studies using sertaconazole for the treatment of otomycosis, some of which are *in vitro* studies. In a study carried out by Moharram AM et al., sertaconazole was added to another antifungal agent, defining fungal species involved in otomycosis as "non-sensitive" to sertaconazole, compared to other medications, such as clotrimazole, terbinafine, or clove oil (19). Furthermore, there are studies in the literature comparing sertaconazole cream with clotrimazole cream in seborrheic dermatitis. Based on the results of a study by Alirezaei et al. (21), the frequency of complications and recurrence of the disease in patients treated with sertaconazole was comparable to the treatment with clotrimazole. However, sertaconazole was found to be more effective, achieving more satisfaction rates among patients (21).

In a study performed by Sumbria D. et al. in 2019, in India, sertaconazole was given to 25 patients, and all became symptom-free at the end of the second week (18). In this study, the microbiology of the species and drug sensitivity were randomly investigated *in vitro*, and the groups of patients (every group had 25 cases) received different antifungal medications (i.e., lotions, gels, and creams, and one group received sertaconazole 2% cream), followed by the investigation of the clinical treatment outcomes. Most of the cultured organisms were sensitive to luliconazole, sertaconazole, and terbinafine with few variations, while fluconazole, clotrimazole, and a combination of 1% clotrimazole, chloramphenicol, and beclomethasone were less effective (18). However, in this study, consistent with our results, sertaconazole revealed a promising response to the treatment rates in the management of otomycosis (18). Nonetheless, our study was more structured and was conducted on a larger sample size, compared to the study by Sumbria et al. (18) in comparing the clinical outcomes of sertaconazole cream treatment with placebo and two other commonly prescribed antifungal creams. 

 The patients were also evaluated three times after the initial treatment (at the end of the first, second, and fourth weeks) for all four major clinical presentations in the present study, while in a study conducted by Alnawaish et al., the evaluations lasted only for the first and second weeks (12). In our study, the rate of otorrhea reached the lowest levels on the fourth week in groups A, B, and C, revealing no differences in applying the three antifungal agents for this particular symptom. In addition, as presented in Figure 2, significant therapeutic outcomes were obtained for miconazole 2% cream, compared to clotrimazole and sertaconazole 2% creams, and the outcomes were clearly better than those in the placebo. On the other hand, considering the complete and partial responses together as the positive response to treatment, the sertaconazole group showed a 96.43% response rate that is comparable with, and even better than that of miconazole and clotrimazole creams, and significantly better than the response rate of placebo. Responding to the treatment in patients taking placebo (10 ears on the first week, 12 ears on the second week, and 10 ears on the fourth week), was probably due to doing complementary treatments (i.e., debridement of EAC, taking acetic acid 2% at the time of debridement, and keeping the ears dry). Any adverse reactions were not observed in our patients during the study in different groups. Furthermore, the patients did not need to apply ear drops several times each day and/or for long-term durations. This may be encouraging for ENT specialists and otologists for selecting anti-fungal creams instead of otic drops in the treatment of otomycosis patients. However, these cases must have intact tympanic membrane and must not have hypersensitivity to the azole drugs. There were no prominent limitations in our study, and the failure rates of attending the follow-up sessions were relatively acceptable. Probably, the implementation of fungal culture before and after the study made it more robust; however, it was attempted to evaluate the "clinical" effects of these antifungal creams. Nevertheless, future studies with larger sample sizes, with or without fungal cultures, in other patient groups (patients with chronic otitis media and perforated tympanic membrane), and other countries are recommended.

## Conclusion

Considering both complete and partial responses together, the sertaconazole group showed a 96.43% response rate. According to our findings, completing the treatment course of otomycosis with sertaconazole, miconazole, clotrimazole (2%) creams revealed acceptable clinical outcomes, compared to placebo. However, it is recommended that future studies be conducted with larger sample sizes and in other countries worldwide.
